# Discovery and structural characterization of the D-box, a conserved TonB motif that couples an inner-membrane motor to outer-membrane transport

**DOI:** 10.1016/j.jbc.2024.105723

**Published:** 2024-02-02

**Authors:** Patrick J. Loll, Kimberly C. Grasty, David D. Shultis, Nicholas J. Guzman, Michael C. Wiener

**Affiliations:** 1 Department of Biochemistry and Molecular Biology, Drexel University College of Medicine, Philadelphia, Pennsylvania, USA; 2 Department of Molecular Physiology and Biological Physics, University of Virginia, Charlottesville, Virginia, USA

**Keywords:** antibiotic resistance, ExbBD, Gram-negative bacteria, TonB, TonB-dependent transport, proton motive force, membrane transport, outer membrane, plasma membrane, siderophore

## Abstract

Gram-negative bacteria use TonB-dependent transport to take up nutrients from the external environment, employing the Ton complex to import a variety of nutrients that are either scarce or too large to cross the outer membrane unaided. The Ton complex contains an inner-membrane motor (ExbBD) that generates force, as well as nutrient-specific transport proteins on the outer membrane. These two components are coupled by TonB, which transmits the force from the inner to the outer membrane. TonB contains an N-terminus anchored in the inner membrane, a C-terminal domain that binds the outer-membrane transporter, and a proline-rich linker connecting the two. While much is known about the interaction between TonB and outer-membrane transporters, the critical interface between TonB and ExbBD is less well understood. Here, we identify a conserved motif within TonB that we term the D-box, which serves as an attachment point for ExbD. We characterize the interaction between ExbD and the D-box both functionally and structurally, showing that a homodimer of ExbD captures one copy of the D-box peptide *via* beta-strand recruitment. We additionally show that both the D-box motif and ExbD are conserved in a range of Gram-negative bacteria, including members of the ESKAPE group of pathogens. The ExbD:D-box interaction is likely to represent an important aspect of force transduction between the inner and outer membranes. Given that TonB-dependent transport is an important contributor to virulence, this interaction is an intriguing potential target for novel antibacterial therapies.

Gram-negative bacterial pathogens pose a significant public-health threat, as many are intrinsically resistant to commonly used antibiotics, and multidrug-resistant strains are now becoming widespread. Recognizing the severity of this threat, both the Centers for Disease Control and Prevention and the World Health Organization have identified as a critical priority the development of new therapeutics aimed at multidrug-resistant Gram-negative bacterial species (1, 2).

One potential target for new antimicrobials is TonB-dependent transport, an energy-dependent process by which Gram-negative organisms import nutrients from their environment (3, 4). While small molecules (molecular weight < ∼600 Da) can diffuse through outer-membrane porins in an energy-independent manner (5), larger species, as well as nutrients that are scarce in the extracellular milieu, are acquired *via* the TonB-dependent mechanism. Substrates taken up include iron and nickel, carbon sources such as peptides and glycans, and organometallic compounds like heme and vitamin B12. The genes associated with TonB-dependent transport are virulence factors (6), making inhibition of the uptake process an attractive strategy.

TonB-dependent transport relies upon the Ton complex, which harnesses the proton-motive force of the inner membrane to drive energy-dependent nutrient uptake at the outer membrane (7, 8). The Ton complex comprises three main components: an inner-membrane motor, an outer-membrane receptor, and a coupling protein that connects the two (Fig. 1*A*). The motor is the ExbBD complex, in which a pentameric ring of ExbB molecules encircles two copies of the ExbD protein. ExbBD is thought to function as a rotary motor driven by the membrane’s electrochemical gradient (7, 9). Multiple different outer-membrane receptors (known as TonB-dependent transporters, or TBDTs) can participate in the Ton complex; Gram-negative bacteria typically possess anywhere from five to 20 TBDTs, each of which is specific for a given substrate. TBDTs bind substrate with high affinity in an energy-independent manner, after which an energy-dependent transport step is initiated, mediated by the coupling protein TonB. TonB contains a single N-terminal helix embedded in the inner membrane and a small globular C-terminal domain (CTD) that interacts with TBDTs; these two features are connected by a proline-rich linker region that spans the periplasm.

**Figure 1:**
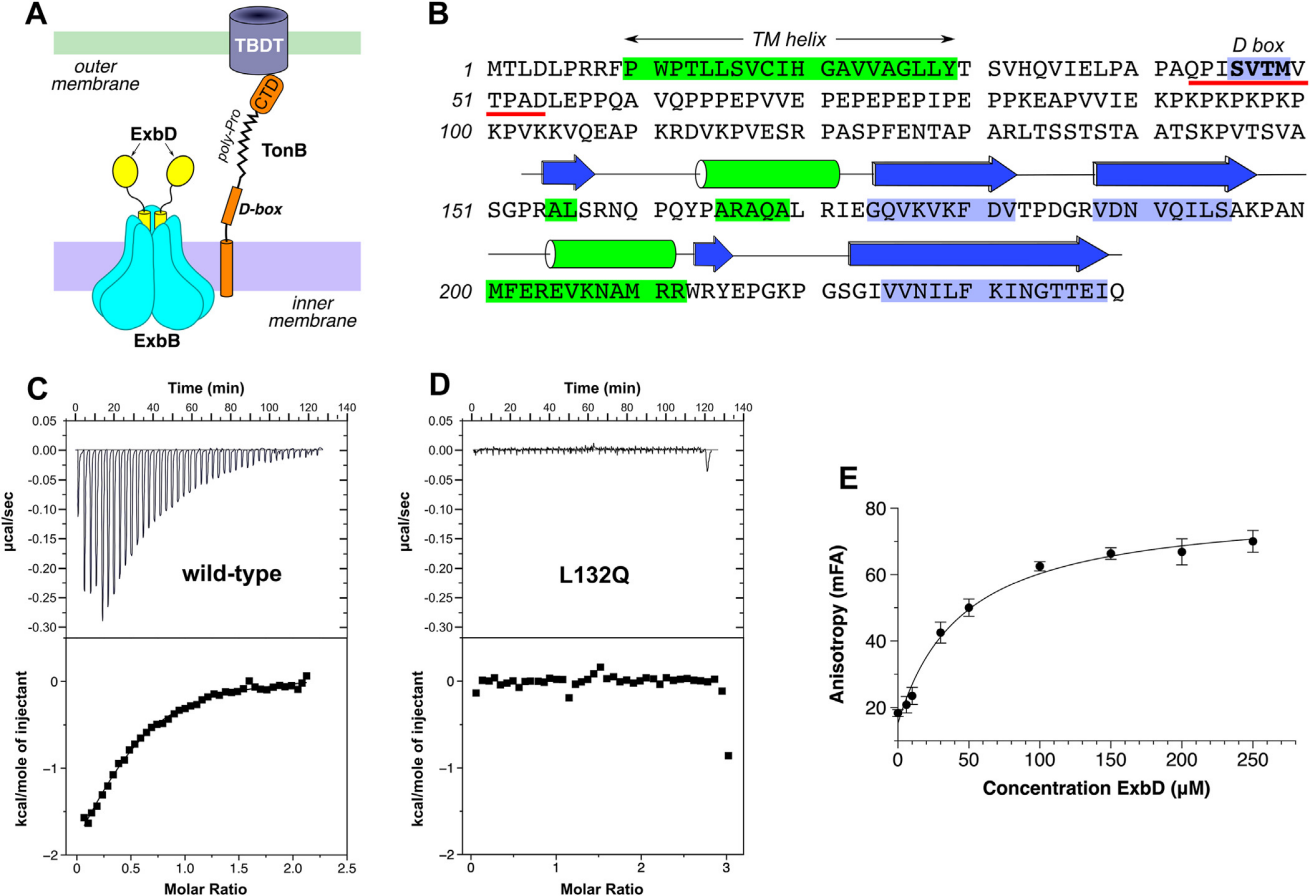
**Recognition of TonB's D-box sequence by ExbD.***A*, schematic view of the Ton complex. *B*, identification of the D-box as a motif with high beta-strand propensity. Shown is the sequence of *E. coli* TonB, with colored highlights indicating secondary-structure predictions from PSIPRED (12). *Blue* indicates predicted beta strands, while *green* indicates alpha helices. The cartoons above the sequence show the actual secondary structure, as determined from structures of the TonB C-terminal domain (10, 11), which is in good agreement with the PSIPRED predictions. The sequence underlined in *red* shows the D-box peptide used for binding assays and crystallization. *C*, ITC data showing binding of a D-box peptide to the purified ExbD_59-141_ protein; estimated K_d_ = 19 ± 1 μM. *D*, the L132Q mutant of ExbD fails to bind the D-box peptide. *E*, fluorescence anisotropy assay demonstrating binding of a fluorescently labeled D-box peptide to the ExbD_59-141_ protein; estimated K_d_ = 45 ± 9 μM. ITC, isothermal titration calorimetry.

TonB’s CTD binds to a periplasm-facing sequence within the TBDT known as the Ton-box, which is exposed when substrate binds to the TBDT at the external surface of the outer membrane. In order for TonB to “energize” substrate transport through the barrel of the TBDT, it must couple to ExbBD in such a way so as to transduce movements of the motor into conformational changes of the TBDT. Presumably ExbBD and TonB must physically interact to complete the energized coupling, but the details of this process have remained unclear. Here, we report a novel interaction between a specific sequence within the TonB linker region and the C-terminal periplasmic domain of ExbD, which we suggest may represent the binding event that is required for energy coupling.

## Results

### Bioinformatics predicts a functional "D-box" motif in TonB

When a TBDT binds its substrate, the Ton-box motif then binds to the CTD of TonB, forming a β-strand that is “recruited” to a pre-existing β-sheet within the CTD (10, 11). *In silico* analyses of Ton-box sequences from various TBDTs indicate that they possess an intrinsic propensity to form β-strands (11), and this propensity is exploited by the CTD when it recruits the Ton-box to join its β-sheet. We wondered whether a similar β-sheet augmentation mechanism underlies ExbBD’s interaction with TonB, perhaps linking a TonB strand to the β-sheet within the periplasmic domain of ExbD. Thus, by analogy to the interaction between the Ton-box motif and the TonB CTD, we posited that TonB might also contain a conserved sequence motif—dubbed the "D-box"—with high β-strand propensity. Indeed, PSIPRED analysis (12) of *Escherichia coli* TonB predicted that the Ser-Val-Thr-Met sequence of residues 46 to 49 forms a β-strand (Fig. 1*B*). This sequence is located immediately downstream of TonB’s membrane anchor and, as such, is well positioned to interact with ExbD’s C-terminal periplasmic domain, which is connected to its membrane-spanning domain by a short linker and is therefore constrained to remain close to the surface of the inner membrane.

### ExbD binds the D-box peptide

**Figure 2:**
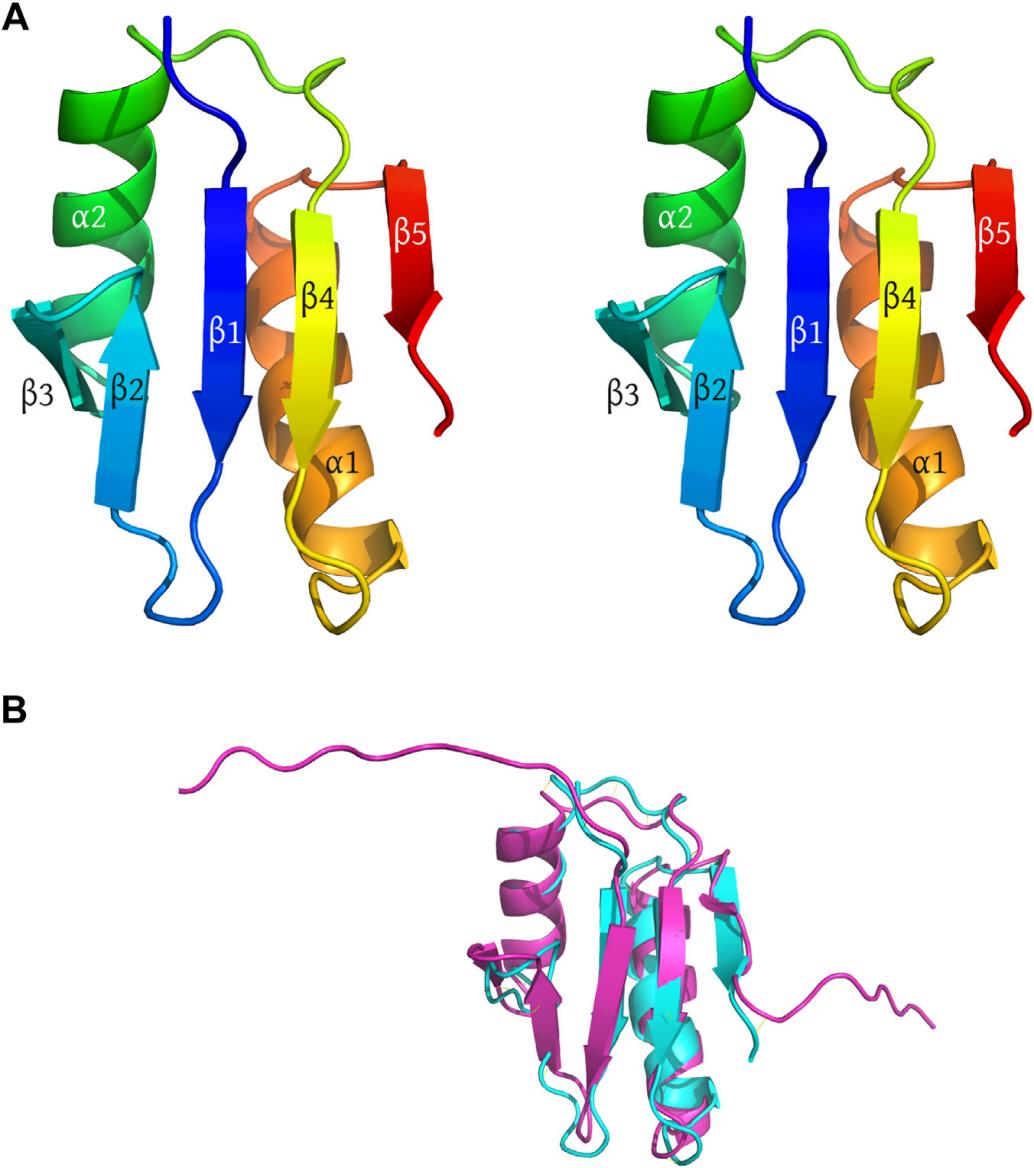
**Structure of the*****E. coli*****ExbD periplasmic domain.***A*, Stereo view of the ExbD periplasmic domain (residues 59–141). The sequence is colored using a rainbow gradient, ranging from *blue* at the N-terminus to *red* at the C-terminus. The various secondary-structural elements are labeled. *B*, comparison of the crystal structure (*cyan*) with the NMR structure (*magenta*; (13)).

To test the hypothesis that the TonB D-box motif binds to ExbD, we used isothermal titration calorimetry to evaluate binding of a D-box peptide to the purified ExbD periplasmic domain. We used a synthetic peptide corresponding to residues 43 to 54 of *E. coli* TonB, which includes the D-box sequence (Fig. 1). To produce the ExbD periplasmic domain, we engineered an expression construct encoding residues 59 to 144 of the *E. coli* protein, with the domain boundaries being chosen to exclude disordered regions identified in a previous NMR structure ((13); Fig. 2). Calorimetry experiments clearly revealed binding of the D-box peptide to ExbD, with an estimated *K*_d_ of 19 ± 1 μM and a stoichiometry of one D-box to two ExbD molecules (Fig. 1*C*). Importantly, the ExbD L132Q mutation, which has previously been shown to abolish TonB-dependent transport (14), abrogates peptide binding (Fig. 1*D*). We then designed an orthogonal binding assay to confirm these observations, choosing to measure fluorescence anisotropy. Consistent with the isothermal titration calorimetry results, a fluorophore-labeled D-box peptide was also found to bind to the purified ExbD periplasmic domain (Fig. 1*E*).

### Structure of the complex of ExbD with the D-box

We next sought to elucidate the structural basis for D-box binding by ExbD. The ExbD periplasmic domain alone yielded no crystals, even when high concentrations of protein were used (data not shown); however, crystals were readily obtained when the periplasmic domain was mixed with the D-box peptide. Two different crystal forms were obtained, both diffracting to approximately 1.4 Å (Table 1); in each, the asymmetric unit comprises a 2:1 ExbD:D-box complex. The complex adopts essentially identical conformations in the two crystal forms (RMSD = 0.68 Å for all Cα positions).

**Table 1:** Data collection and refinement statistics

Data collection statistics	Orthorhombic	Tetragonal	Pt derivative
PDB ID	8VGC	8VGD	—
Diffraction source	Beamline 17-ID-1, NSLS-II	Beamline 17-ID-1, NSLS-II	Beamline 24-ID-C, APS
Wavelength (Å)	0.92011	0.92011	1.0718
Temperature (K)	100	100	100
Detector	Eiger 9M	Eiger 9M	Dectris Pilatus 6 MF
Resolution range (Å)^a^	24.63–1.42 (1.47–1.42)	26.40–1.42 (1.47–1.42)	47.0–2.30 (2.38–2.30)
Spacegroup	*P*2_1_2_1_2_1_	*P*4_1_	*P*2_1_2_1_2_1_
Unit cell			
a, b, c (Å)	49.26, 60.64, 73.91	49.01, 49.01, 62.64	49.96, 60.92, 73.82
α, β, γ (°)	90.0, 90.0, 90.0	90.0, 90.0, 90.0	90.0, 90.0, 90.0
Total number of observations	569,152 (58,417)	392,947(29,191)	150,168 (13,631)
Number of unique reflections	42,453 (4172)	27,989 (2029)	19,315 (1959)
Average multiplicity	13.4 (14.0)	14.0 (14.4)	7.8 (7.0)
Completeness (%)	99.9 (99.9)	100.0 (100.0)	99.96 (99.85)
Mean I/sigma(I)	17.4 (1.2)	13.6 (0.7)	31.5 (8.4)
Estimated Wilson B-factor (Å^2^)	27.8	26.6	54.0
R-merge^b^	0.069 (2.49)	0.080 (4.611)	0.035(0.225)
R-meas^c^	0.072 (2.58)	0.083 (4.781)	0.038 (0.038)
R-pim^d^	0.020 (0.681)	0.022 (1.260)	0.013 (0.091)
CC_1/2_^e^	0.998 (0.446)	0.998 (0.313)	0.999 (0.978)
CC_1/2,anom_^e^	—	—	0.971 (0.523)
Refinement and model statistics			
Resolution range (Å)	24.63–1.42 (1.46–1.42)	26.39–1.42 (1.47–1.42)	—
Number of reflections used	40,450 (2842)	26,548 (2622)	—
Reflections used for R-free	1999 (141)	1395 (141)	—
Rwork	0.210 (0.372)	0.187 (0.421)	—
Rfree	0.221 (0.493)	0.204 (0.410)	—
Number of non-hydrogen atoms			
Protein + peptide	1221	1224	—
Solvent	86	50	—
Average B-factor (Å^2^)			—
Protein	39.2	49.8	—
D-box peptide	43.3	58.7	—
Solvent	40.8	41.7	—
RMS deviations from ideality			
Bonds (Å)	0.013	0.016	—
Angles (°)	1.35	1.50	—
Residue distribution in Ramachandran plot			
Most favored region (%)	100.0	98.7	—
Allowed (%)	0	1.3	—
Outliers (%)	0	0	—
Clashscore	3.5	6.4	—

a Values in parentheses refer to the highest resolution shell.

b R_merge_ is calculated by the equation R_merge_ = Σ_hkl_ Σ_i_ |I_i_(hkl) − <I(hkl)>|/Σ_hkl_ Σ_i_ I_i_(hkl), where I_i_(hkl) is the i^th^ measurement.

c R_meas_ (or redundancy-independent R_merge_) is calculated by the equation R_meas_ = Σ_hkl_[N/(N − 1)]^½^ Σ_i_ |I_i_(hkl) − <I(hkl)>|/Σ_hkl_ Σ_i_ I_i_(hkl), where I_i_(hkl) is the i^th^ measurement and N is the redundancy of each unique reflection hkl (44).

d R_pim_ is calculated by the equation R_pim_ = Σ_hkl_[1/(N − 1)]^½^ Σ_i_ |I_i_(hkl) − <I(hkl)>|/Σ_hkl_ Σ_i_ I_i_(hkl), where I_i_(hkl) is the i^th^ measurement and N is the redundancy of each unique reflection hkl (45).

e CC_1/2_ is the correlation coefficient between two randomly chosen half data sets (46).

**Figure 3:**
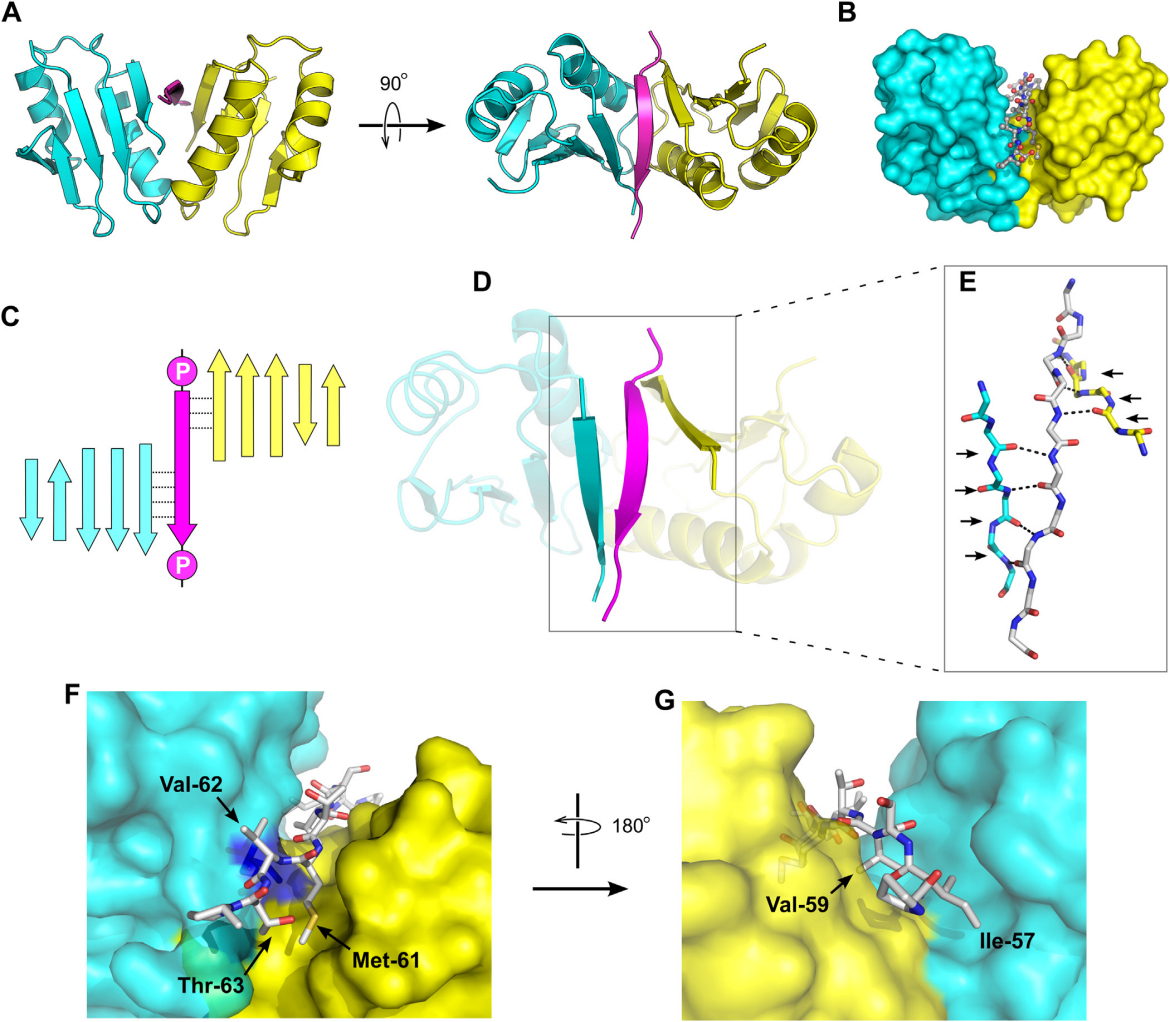
**Recognition of the D-box by the ExbD periplasmic domain.***A*, orthogonal views of the ExbD periplasmic domain dimer in complex with the D-box peptide. Chain A of ExbD is shown in *cyan*, while chain B is shown in *yellow*; the D-box peptide is colored *magenta*. *B*, a surface representation for ExbD emphasizes the narrow groove formed between the two protomers. The D-box peptide is shown in ball-and-stick representation. *C*, schematic representation of how the beta strand formed by the D-box peptide (*magenta*) is recruited both to the beta sheet of the A chain (*cyan*) and to the beta sheet of the B chain (*yellow*). *D*, actual view of the complex structure, highlighting the D-box strand and its neighboring strands in the two ExbD protomers. Color scheme is the same as in the preceding panel. *E*, backbone-backbone hydrogen bonds connecting the D-box strand to its neighboring strands in ExbD. *Right-pointing arrows* show H-bonds corresponding to a parallel-type beta interaction, while *left-pointing arrows* show an antiparallel beta interaction. *F* and *G*, two views of the ExbD complex with the D-box peptide, highlighting the insertion of hydrophobic side chains from the D-box into complementary pockets formed by the protein dimer. In panels *E*–*G*, carbon atoms of the D-box peptide are shown in *gray*.

The crystal structures reveal an overall fold for the ExbD protein that is in good agreement with the previously reported solution structure ((13); Fig. 2). Each ExbD monomer contains a five-stranded sheet, with two helices packed against one face of that sheet; two ExbD monomers assemble into a dimer with approximate two-fold symmetry. The two protomers contact one another *via* the loops connecting strand three and helix 2, imparting a V-shape to the dimer, with a deep groove between the two halves. The D-box peptide binds within this groove (Fig. 3, *A* and *B*) and, as hypothesized, assumes an extended conformation, forming a strand that is recruited to the edges of the beta sheets of both ExbD monomers. Residues 60 to 62 of the peptide interact with residues 130 to 134 of one ExbD chain (Chain A), forming a parallel pattern of inter-strand hydrogen bonds, while residues 57 to 59 of the peptide interact with residues 130 to 132 of the other ExbD chain (Chain B), forming an anti-parallel hydrogen-bonding pattern (Fig. 3, *C*–*E*). Thus, a single D-box strand is simultaneously recruited to the edges of two different beta sheets, one from each of the ExbD protomers. Both ends of the D-box region are flanked by prolines, which effectively delimits the portion of TonB that can be captured by β-strand recruitment.

Because the D-box peptide adopts an extended conformation, the side chains of alternating residues point either up (toward solvent) or down (into the interface with ExbD). This structural arrangement is reflected in a pattern of alternating polar and non-polar residues within the D-box sequence. In particular, the side chains of Ile-57, Val-59, and Met-61 all point downward and are captured within hydrophobic cavities formed in the cleft between the two ExbD protomers (Fig. 3, *F* and *G*). These side chains are essentially completely buried in the complex, losing between 95% and 100% of their solvent-accessible area upon complex formation. Met-61 lies at the C-terminal end of the D-box, and the hydrophobic pocket containing its side chain is partially formed by the side chain of Leu-132, offering an explanation for why the L132Q mutant fails to bind the D-box. Solvent access to this pocket is largely blocked by the side chain of Thr-63 of the D-box, completing burial of Met-61.

**Figure 4:**
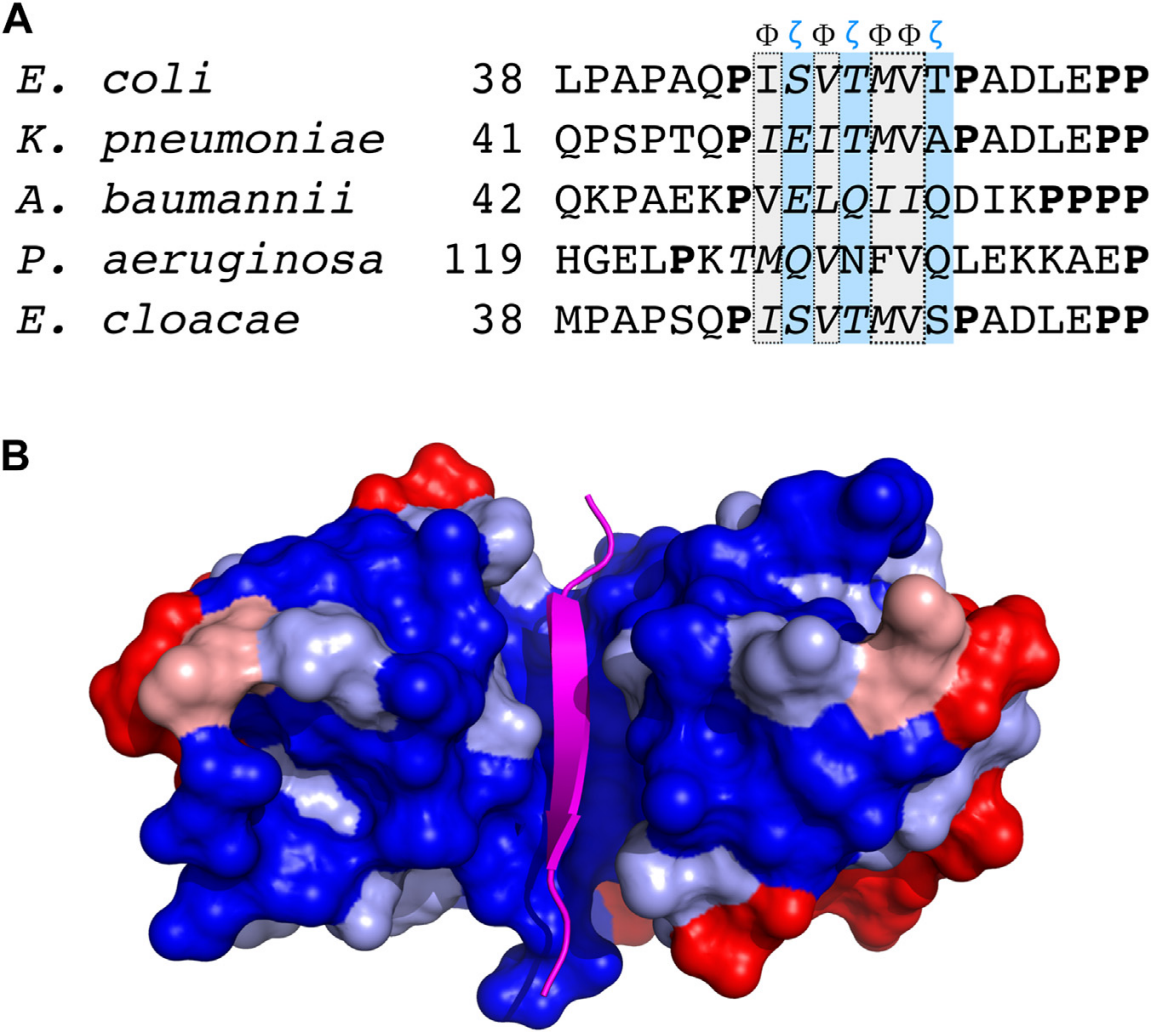
**ExbD and D-box structures are conserved among Gram-negative pathogens.***A*, clustal Omega alignment of TonB sequences from *E. coli* and the Gram-negative ESKAPE pathogens (36). Shown here is the region corresponding to the D-box (full alignment is shown in Fig. S3). The pattern of hydrophobic residues (Φ; *gray*) and hydrophilic residues (ζ; *blue*) is highlighted. The italicized residues are predicted to form beta strands by PSIPRED. Prolines flanking the D-box are shown in bold face. *B*, sequence conservation in ExbD orthologs. ExbD sequences from *E. coli* and the four Gram-negative ESKAPE pathogens were aligned, and sequence conservation was mapped onto the structure of the *E. coli* ExbD:D-box complex. Color code: *Dark blue*, complete sequence conservation; *light blue*, conservation of strongly similar amino acids; *pink*, conservation of weakly similar amino acids; *red*, no sequence conservation. The D-box peptide is shown in *magenta*.

The residue following Met-61 in the D-box sequence, Val-62, is also hydrophobic, breaking the pattern of alternating hydrophobic and hydrophilic amino acids; this departure can be rationalized structurally because the valine side chain packs into a shallow hydrophobic pocket on the surface of chain A. Overall, the sequence motif in the D-box recognized by ExbD takes the form Φ**ζ**Φ**ζ**ΦΦ**ζ**, where Φ and **ζ** denote hydrophobic and hydrophilic residues, respectively; this motif is flanked on both sides by proline residues (Fig. 4).

Apart from the inter-strand hydrogen bonding described earlier, there are no direct polar contacts between the D-box peptide and either the A or B chain. However, a network of water molecules bridges the peptide with both ExbD molecules. For the A chain, waters connect the protein backbone of Tyr-127, Leu-128, and Ile-130 to backbone and side-chain groups of Ser-58 and Thr-60 in the D-box. For the B chain, three additional water-mediated contacts bridge backbone atoms of Leu-128, Ile-130, and Leu-132 on the B chain with backbone and side-chain atoms on Gln-55, Ser-58, and Met-61 in the D-box.

## Discussion

The current model for TonB-dependent transport postulates that TonB transforms movement of the inner-membrane motor ExbBD into a force that is ultimately applied to the outer-membrane TBDT. Presumably the motor must directly bind TonB in order to transmit force across the periplasm, but details have been lacking. Here, we identify a novel interaction between the periplasmic domain of ExbD and a conserved sequence in TonB that we term the D-box. Our structural characterization of the ExbD:D-box complex reveals that two copies of the ExbD periplasmic domain assemble around one copy of the D-box sequence. Essentially identical structures are seen for the complex in two different crystal forms; in addition, these structures are highly similar to one recently published, which describes a 2:1 complex of the ExbD periplasmic domain with a 23-mer TonB peptide that includes the D-box sequence (15). Importantly, the L132Q mutation in ExbD, which destroys TonB-dependent transport *in vivo* (14), also abolishes ExbD’s ability to recognize the D-box *in vitro*, in a manner that is clearly explained by the structure. Hence, D-box recognition is linked to the function of the Ton complex.

The ExbBD motor contains two copies of ExbD, enclosed within a pentameric ring of ExbB molecules (16, 17, 18). The 2:1 stoichiometry of the ExbD:D-box complex suggests that the two copies of ExbD within a single motor complex cooperate to bind the D-box sequence. Currently available structures of the ExbBD motor do not include TonB, and in these structures, the periplasmic domains of ExbD remain disordered (16, 17, 18). This suggests that ExbD binding to the D-box, with concomitant dimerization of periplasmic domains of ExbD, could be a significant, and perhaps even essential, aspect of force transduction between the inner-membrane ExbBD motor and the outer-membrane TBDT.

The region on TonB that serves as the “handle” *via* which ExbBD exerts force has not been clearly defined. The membrane-embedded N-terminal helix of TonB has previously been shown to interact with ExbB (19, 20, 21, 22, 23), and we now identify a second point of contact between the D-box and the periplasmic domains of ExbD. Disruption of either interaction disrupts TonB-dependent transport (14, 22, 23), but the specific molecular roles played by each interaction remain speculative. However, simple structural arguments suggest that the ExbD:D-box interaction is the one responsible for applying tension to TonB. The components of the ExbBD motor are localized within and close to the inner membrane; in contrast, TonB is a long molecule, most of which is thought to adopt a stiff Type-II polyproline helix extending across the periplasm (24, 25, 26). This implies that only the N-terminal portion of TonB will be found in close proximity to ExbBD, and this is exactly where the D-box motif is found, immediately upstream of the putative polyproline helix. This polyproline region is rigid, which is necessary for its proper function (27). Hence, the D-box is directly connected to the CTD *via* a mechanically rigid link and as such is ideally positioned to act as the coupling that transmits force from the motor to TonB.

If ExbD does in fact exert force *via* the D-box, then ExbB’s interaction with TonB’s N-terminal helix may play a role unrelated to force generation, for example, localizing TonB to the vicinity of the motor so that the D-box can engage with ExbD. Interestingly, the ExbB-TonB interaction is mirrored by a similar one between TolA and TolQ, both of which involve similar “SHLS” sequence motifs within the transmembrane helices of TonB and TolA (20, 28). The periplasmic domain of TolA also contains a sequence similar to TonB’s D-box (Fig. S5). Because the periplasmic domain of TolR adopts a fold that is highly similar to that of ExbD (29), it seems reasonable that TolR could dimerize and bind the D-box-like motif in TolA in a manner analogous to the ExbD:D-box interaction. This model explains TolQR’s ability to partially complement deletion of ExbBD (30, 31): In the absence of ExbBD, TonB might be able to utilize force generation by TolQR to drive nutrient uptake at the outer membrane, by first binding to TolQ *via* its SHLS sequence, followed by binding of its D-box to TolR.

Finally, we searched for D-box motifs in Gram-negative organisms other than *E. coli*. Focusing on the Gram-negative members of the ESKAPE group of pathogens (32), we found that TonB proteins in all the organisms considered contained a region with high β-strand propensity in approximately the same position occupied by the D-box in the *E. coli* sequence (Supporting information, Fig. S3). Furthermore, when the TonB sequences from these different organisms are aligned with the *E. coli* sequence, they all display the Φ**ζ**Φ**ζ**ΦΦ**ζ** pattern within the D-box that was described earlier (Fig. 4). In addition, the sequences of these organisms’ ExbD proteins are highly similar, with sequence identities with the *E. coli* protein that range from 65% to 98.6% (Fig. S4). Notably, the regions of ExbD that mediate dimerization and interaction with the D-box are particularly highly conserved (Fig. 4*B*). Taken together, these observations strongly suggest that the ExbD:D-box interaction is conserved in other Gram-negative species, including clinically significant pathogens.

## Experimental procedures

### Construct preparation

The coding sequence for *E. coli* ExbD residues 59 to 141 was codon optimized using the JCat tool (33). The gene was then synthesized (Twist Bioscience), along with flanking regions that allowed it to be assembled into the BseRI site of the pETHSUL expression plasmid (34), using the NEBuilder HiFi DNA assembly kit (New England Biolabs). The resulting construct encodes His_6_-SUMO fused to the N-terminus of the ExbD periplasmic domain, with a single glycine inserted between the SUMO and ExbD sequences. The purified ExbD protein produced after proteolytic removal of the SUMO tag contains a single glycine residue upstream of Arg-59.

### Expression and purification

The ExbD periplasmic domain was expressed in *E. coli* BL21(DE3) and purified by subtractive immobilized-metal ion chromatography followed by size-exclusion. Details are given in the Supporting information.

### Isothermal titration calorimetry

The D-box peptide, corresponding to residues 43 to 54 of TonB (QPISVTMVTPAD), was synthesized by VWR Scientific. Purified wild-type and L132Q ExbD proteins and the D-box peptide were separately dialyzed overnight against 4 L of 10 mM MES pH 5.3, 60 mM NaCl at 277 K. Using a MicroCal calorimeter, the D-box peptide (1 mM) was injected into a 100-μM solution of ExbD protein at 288 K; the first injection was not used in the binding energetics determination. A binding isotherm was fit for the D-box peptide binding to wild-type protein using a two-state binding model. In contrast, no interaction was detected between the L132Q mutant and the D-box peptide.

### Fluorescence anisotropy binding assay

A synthetic peptide having the sequence QPISVTMVTPADC was purchased from Biomatik Corporation. Peptide measuring 2.5 mg was dissolved in 0.5 ml of 100 mM HEPES (4-(2-hydroxyethyl)piperazine-1-ethanesulfonic acid) pH 7.5 and 1 mM ethylenediaminetetraacetic acid, and the peptide solution was added to 0.5 ml of beads bearing immobilized tris-(2-carboxyethyl)phosphine (Thermo-Pierce). The tube was rotated at room temperature for 1 hour, after which the beads were removed by filtration. A 50 mg/ml solution of AF488-maleimide (BP-Fluor488, cat. no. BP-25506, Broad Pharm) was prepared in dimethyl sulfoxide. One hundred microliters of the dye solution was added to the peptide, and the mixture was incubated at room temperature, protected from light, for 2 hours. The reaction was then quenched by addition of 5 μl of 2-mercaptoethanol, followed by an additional one-hour incubation at room temperature. Labeled peptide was purified on a C18 column (Ultrasphere 5-micron ODS, 1.0 × 25 cm; Hichrom Ltd), using a 5% to 100% gradient acetonitrile in a mobile phase containing 0.2% formic acid. Purified labeled peptide was then lyophilized and redissolved in a small volume of 40 mM Tris pH 8.0 and 150 mM NaCl.

Fluorescence anisotropy experiments were conducted in 100-μL volumes containing 100-nM AF488-labeled peptide in 40 mM Tris pH 8.0 buffer containing 150 mM NaCl, using black 96-well half-area plates (Corning # 3993). Anisotropy measurements were conducted at room temperature using a Tecan Spark microplate reader. Samples were excited at 480 nm and emission read at 530 nm, using a 5-nm bandpass for both emission and excitation. A 510-nm dichroic mirror was used to condition the emitted signal. K_d_ values were estimated by fitting the following expression to the data:

$$A\;=\;A_{0}\;+\;\left(A_{\max}\;-\;A_{0}\right)∙\left[\frac{\left(K_{D}\;+\;L_{t}\;+\;P_{t}\right)\;-\;\sqrt{{\left(K_{D}\;+\;L_{t}\;+\;P_{t}\right)}^{2}\;-\;4P_{t}L_{t}}}{2P_{t}}\right]$$

where A denotes anisotropy, and L_t_ and P_t_ are the total concentrations of peptide and protein, respectively.

### Crystallization and data collection

Detailed methods are given in the Supporting information. Briefly, crystals of the complex of ExbD with the D-box peptide were grown from ammonium sulfate solutions, optimizing conditions discovered from commercial screens; both an orthorhombic and a tetragonal crystal form were obtained under slightly different final conditions. The structure of the orthorhombic form was determined by single-isomorphous replacement with anomalous scattering, using a platinum derivative prepared with K_2_PtCl_4_. This structure was then used as a probe to determine the structure of the tetragonal form *via* molecular replacement. Models were refined with Phenix (35) and deposited in the Protein Data Bank with accession numbers 8VGC and 8VGD. Data collection and refinement statistics are shown in Table 1.

## Data availability

All data are provided in the manuscript with the following exceptions: Example data for the purification of the ExbD periplasmic domain are shown in the Supporting information, Fig. S1; alignment data for TonB and ExbD sequences are shown in Supporting information Figs. S3 and S4, respectively; and accession numbers for protein sequences used in alignments are shown in Supporting information Table S1. Coordinates and structure factors for the orthorhombic and tetragonal models are deposited with the Protein Data Bank, accession codes 8VGC and 8VGD, respectively. Raw diffraction data have been archived with the Zenodo repository and may be accessed at https://doi.org/10.5281/zenodo.10574019 (orthorhombic data set) and https://doi.org/10.5281/zenodo.10574068 (tetragonal data set).

## Supporting information

This article contains supporting information (12, 34, 36, 37, 38, 39, 40, 41, 42, 43).

## Conflict of interest

The authors declare that they have no conflicts of interest with the contents of this article. One author is an editorial board member for the Journal of Biological Chemistry and was not involved in the editorial review or the decision to publish this article.
