# Supplementary material for: Discovery and structural characterization of the D-box, a conserved TonB motif that couples an inner-membrane motor to outer-membrane transport
Source: J Biol Chem. 2024 Feb 2;300(3):105723. doi: 10.1016/j.jbc.2024.105723 (PMC10907165; doi:10.1016/j.jbc.2024.105723)
Supplement: Supporting information [file mmc1.docx]

***Supporting Information***

**Discovery and structural characterization of the D-box, a conserved TonB motif that couples an inner-membrane motor to outer-membrane transport**

Patrick J. Loll, Kimberly C. Grasty, David D. Shultis, Nicholas J. Guzman, and Michael C. Wiener

***List of Contents***

***Detailed methods:*** Expression and purification S2

***Fig. S1.*** Purification of the ExbD periplasmic domain S3

***Detailed methods:*** Crystallization and structure determination S4

***Fig. S2.*** Representative electron density S5

***Fig. S3.*** Alignment of TonB sequences from Gram-negative pathogens S6

***Fig. S4.*** Alignment of ExbD sequences from Gram-negative pathogens S7

***Table S1.*** Accession numbers for protein sequences used in alignments S7

***Fig. S5.*** A potential D-box sequence in *E. coli* TolA S8

***References*** S9

***ExbD expression and purification.*** The expression plasmid for the ExbD periplasmic domain was transformed into *E. coli* BL21(DE3). Starter cultures were grown overnight at 37° in LB media containing 100 μg/mL ampicillin. 1 mL of this overnight culture was inoculated into 500 mL of self-inducing media containing 100 μg/mL ampicillin at 25 °C, and cultures were shaken for 20-24 hours. Cells were harvested and resuspended in 20 mM Tris, pH 7.8 containing 250 mM NaCl, 10 mM imidazole (Buffer A), supplemented with 10 mM MgSO_4_, 2 µg/mL DNase, and 2 µg/mL RNase. The cell suspension was passed three times through an Avestin Emulsiflex C5 cell disrupter. The lysate was spun at 15,000x*g* for 30 minutes, filtered through a 0.45-μm filter, and loaded on to a nickel-charged 5-mL IMAC HP column (Cytiva). The column was subsequently washed with 10-20 CVs of Buffer A before being eluted with 20mM Tris, pH 7.8, 250 mM NaCl, 250 mM imidazole. The protein-containing fractions were pooled, 500 μg of the SUMO hydrolase dtUD1 were added (1), and the solution was dialyzed vs. Buffer A overnight at 4 °C. The following day the dialyzed protein was again loaded onto a 5-mL HiTrap IMAC column equilibrated with Buffer A and the flow-through, containing the ExbD periplasmic domain, was collected. The resulting protein was concentrated to about 5 mL and injected onto a S200 26/60 column (Cytiva) equilibrated with 20 mM Tris, pH7.8, 150 mM NaCl. Fractions containing the protein were pooled, dialyzed into 50 mM MES, pH 5.3, 50 mM NaCl and concentrated to 30 mg/mL for crystallization experiments. Protein concentration was estimated using a BCA assay.


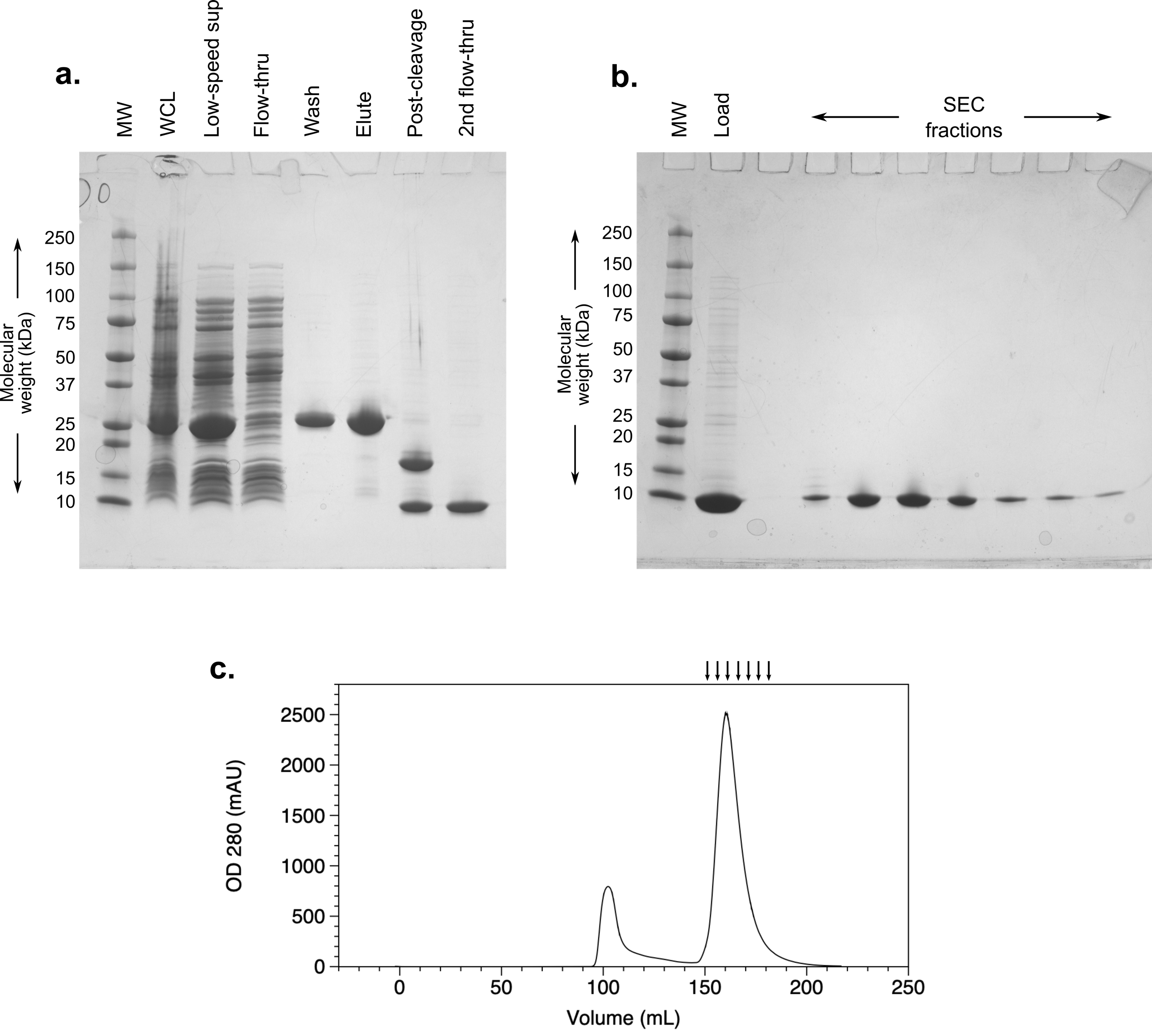


***Figure S1*.** Purification of the ExbD periplasmic domain. (*a*) & (*b*) Coomassie-stained SDS PAGE gels tracking the purification process. (*a*) Subtractive IMAC. WCL, whole-cell lysate; Post-cleavage: Sample after cleavage with the SUMO hydrolase UD1. (*b*) Size-exclusion chromatography of the sample after subtractive IMAC, using a 26/60 Sephacryl S-100 column. Load, sample loaded on the column. Fractions were taken across the major elution peak. (*c*) Chromatogram corresponding the gel in panel *b*. Arrows indicate the fractions shown in the gel.

***Crystallization and structure determination****.* Purified ExbD (30 mg/mL) was mixed in a 2:1 mole ratio with the D-box peptide, giving a final protein concentration of 25.4 mg/mL. The mixture was incubated at 4 °C for 1 hour before being subjected to crystallization screening experiments using the microbatch-under-oil technique (2,3). Initial screening was performed using three commercial screens (Berkeley Screen, PACT+ Screen and Wizard-Classics 1 and 2). Optimized orthorhombic crystals were obtained at 4 °C by either microbatch-under-oil or handing-drop vapor diffusion, using a precipitant solution containing 2 M ammonium sulfate in 0.1 M sodium acetate, pH 4.6. Tetragonal crystals were grown at 4 °C using a precipitant solution containing 2 M ammonium sulfate in 0.1 M sodium citrate, pH 5.8. Derivative crystals for heavy-atom phasing were produced by adding to the hanging drop 1 μL of a solution of 20 mM potassium tetrachloroplatinate in reservoir buffer (2 M ammonium sulfate in 0.1 M sodium acetate, pH 4.6); drops were incubated in the presence of the platinum compound for 5 h at room temperature before mounting.

Crystals were captured in lithographic polymer loops (Mitegen), dragged briefly through Al’s oil or Fomblin oil, and flash-cooled by plunging into liquid N_2_. Diffraction data were collected at beamlines 17-ID-1 of the National Synchrotron Light Source II (NSLS-II) and 24-ID-C of the Advanced Photon Source (APS); data were processed using XDS (4). Two well-diffracting crystal forms were obtained (Table 1), one belonging to an orthorhombic point group, and other to a tetragonal point group. The structure of the orthorhombic crystal form was determined by SIRAS phasing, using the platinum chloride derivative and the Autosol pipeline in Phenix (5). The model was improved by alternating cycles of manual rebuilding in Coot (6) and refinement in Phenix. This model was then used a molecular-replacement probe to determine the structure of ExbD in the remaining tetragonal crystal form, using PHASER (7), after which this structure were refined in Phenix.

In both crystal forms, the asymmetric unit contains an ExbD dimer plus one copy of the D-box peptide. The two ExbD protomers are related by a two-fold axis of symmetry, and the D-box peptide is bound across this symmetry axis. Hence, in principle the peptide could exhibit two-fold rotational disorder. However, for all the crystals analyzed, the electron density supports a single orientation for the peptide, suggesting that the crystal milieu favors assembly of the complex in an ordered fashion (even though the D-box peptide is not involved in any crystal contacts). In the case of the tetragonal structure, the intensity data initially suggested a 422 point group in which the asymmetric unit contained one ExbD molecule; this would require that the peptide lie on a crystallographic two-fold axis, and thus be present in two orientations. However, refinement of the model in space group *P*4_1_2_1_2 stalled with high R-values. Hence, the data were reprocessed using the lower-symmetry space group *P*4_1_, corresponding to one ExbD dimer + one D-box peptide per asymmetric unit. Using a single orientation for the peptide, refinement of this model converged without problems.

In the final refined models, all chains show good density for at least residues 63-133 of ExbD and residues 55-63 of the D-box peptide; some chains show a few additional residues at either end of the chain. Data collection, refinement statistics, and PDB IDs are shown in Table 1. Representative electron density for the final model is shown in Figure S2.


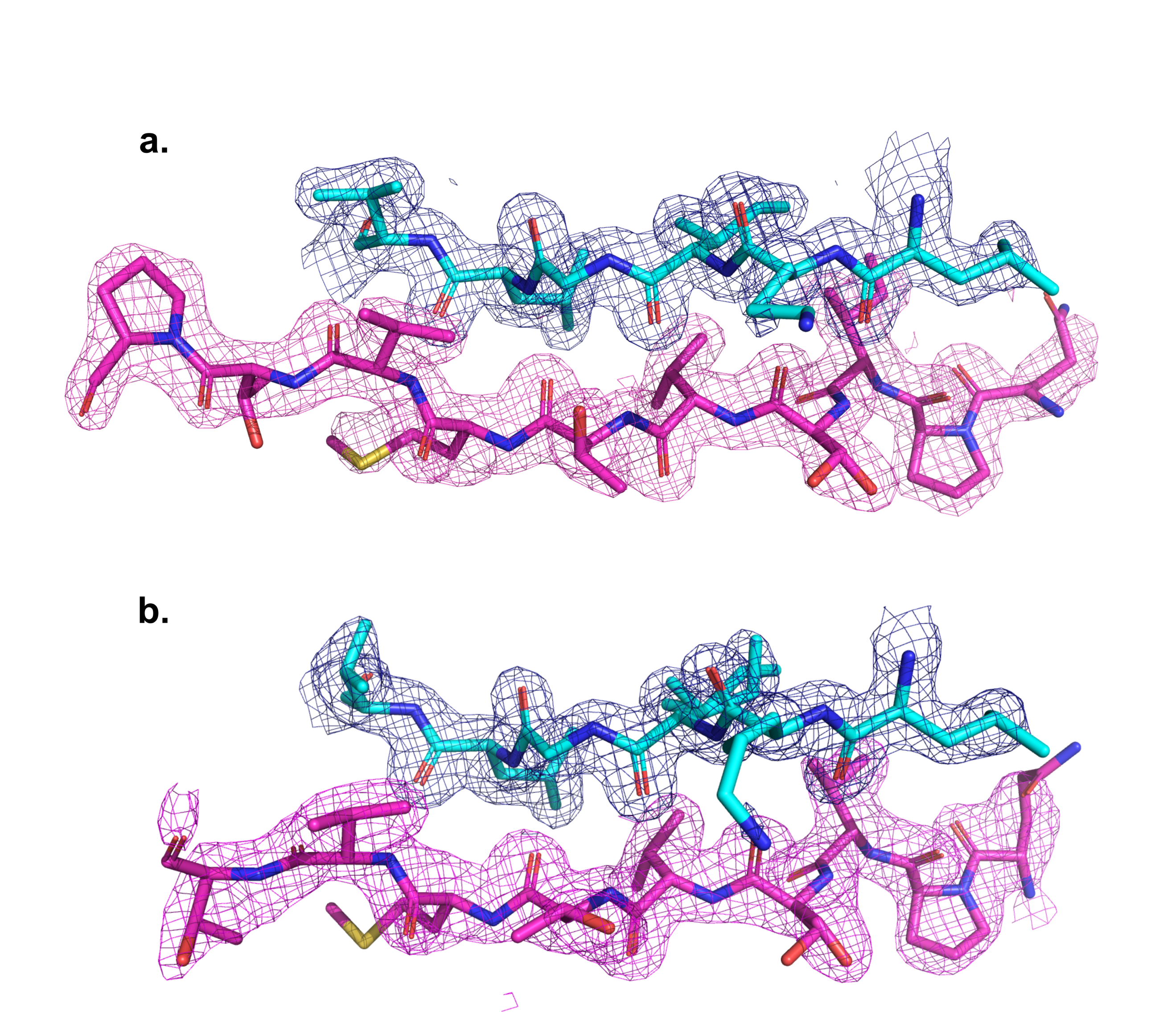


***Figure S2.*** Representative electron density. Shown are portions of the 2Fo-Fc map from the final refined structures, contoured at 1.3 σ. (a) Orthorhombic crystal form. (b) Tetragonal crystal form. In both images, the magenta sticks show the D-box peptide, while the cyan sticks show strand β5 from the A chain of ExbD. This portion of the structure shows parallel strand recruitment to the edge of the beta sheet in chain A. Map was displayed with a carve radius of 2 Å.


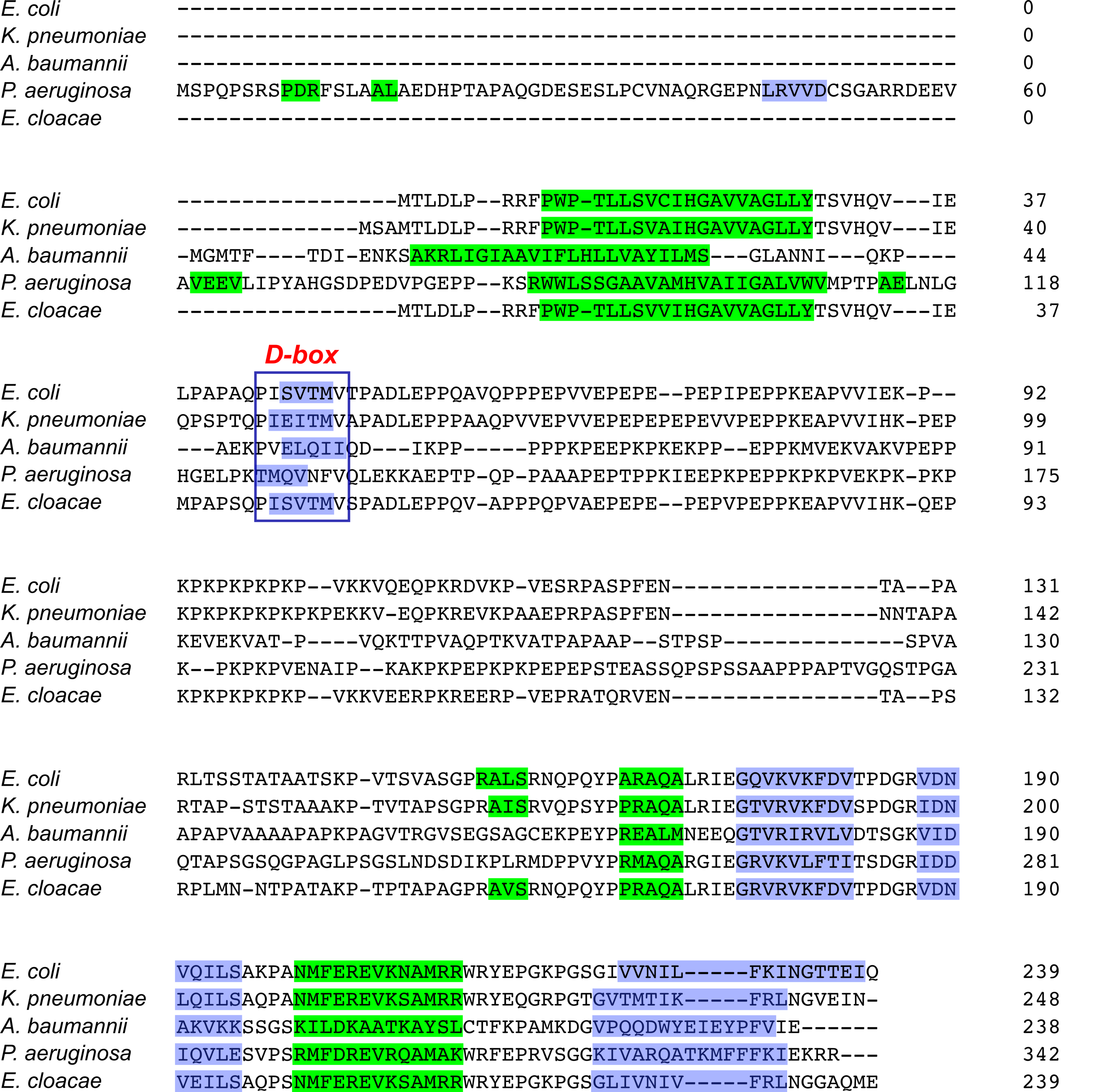


***Figure S3.*** Clustal Omega alignment of TonB proteins from *E. coli* and the Gram-negative ESKAPE pathogens (8). Color coding is the same as used in Fig. 1: Green, alpha helices predicted by PSIPRED; blue, beta strands predicted by PSIPRED (9). The position of the D-box motif is indicated.

60 70 80 90 100 110 120 130 140

| | | | | | | | |

*E. coli* RPEKPVYLSVKADNSMFIGNDPVTDETMI-TALNALTEGKKDTTIFFRADKTVDYETLMKVMDTLHQAGYLKIGLVGEETAKAK

*K. pneumoniae*  RPEKPIYLSVKADKSMFLGNDPVTEANMI-NALNSLTAGKKDTTVFFRADKTVDYETMMKVMDTLHQAGYLKIGLVGEETVKAK

*A. baumannii* RPEKPVYLSVKADNSMFIGNDPVTDETMI-TALNALTEGKKDTTIFFRADKTVDYETLMKVMDTLHQAGYLKIGLVGEETAKAK

*P. aeruginosa* RPEKPVFVSVKADQKLYVGDDQVPAPDQLGPMLDAKTKGDKETTIFFQADKGVDYGDLMEVMNNMRSAGYLKVGLVGLETAAKK

*E. cloacae* RPEKPIYLSVKADNSMFIGNDPVTEETMI-NQLNAVTEGKKDTTIFFRADKTVDYETMMKVMDSLHQAGYLKIGLVGEEVAKAK

*****:::*****:.:::*:* * : *:: * *.*:**:**:*** *** :*:**:.::.*****:**** *.. *

|  | ***Sequence identity (similarity)*** |
| --- | --- |
| *E. coli* | 100 (100) |
| *K. pneumoniae* | 88.7 (94.3) |
| *A. baumannii* | 98.6 (98.6) |
| *P. aeruginosa* | 65.0 (79.7) |
| *E. cloacae* | 91.5 (96.5) |

***Figure S4.*** Top, Clustal Omega multi-sequence alignment of ExbD proteins from *E. coli* and the Gram-negative ESKAPE pathogens. Numbering shown is for the *E. coli* protein. Consensus symbols: asterisk, complete conservation; colon, conservation of strongly similar amino acids; period, conservation of weakly similar amino acids. Below, sequence identity and similarity for ESKAPE-pathogen ExbD sequences as compared with the *E. coli* sequence. Sequences were aligned in a pairwise manner using EMBOSS Needle (10).

| **Organism** | **TonB Sequence Accession No.** | **ExbD Sequence Accession No.** |
| --- | --- | --- |
| *Escherichia coli* | NP_415768.1 | WP_001240712.1 |
| *Klebsiella pneumoniae* | WP_015958608.1 | WP_040169836.1 |
| *Acinetobacter baumannii* | BCA99614.1 | MDC5482191.1 |
| *Pseudomonas aeruginosa* | NP_254218.1 | PPB14291.1 |
| *Enterobacter cloacae* | WP_038419780.1 | MBS5772198.1 |

***Table S1***. Accession numbers for protein sequences used in alignments.


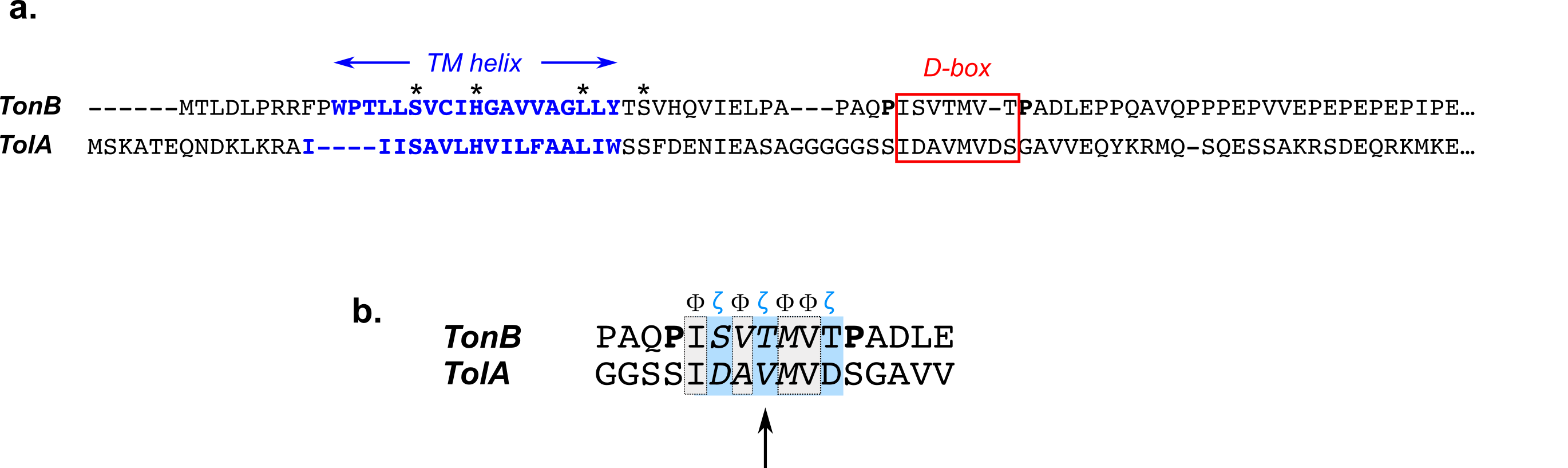


***Figure S5.*** *E. coli* TolA contains a potential D-box sequence. a) Alignment of the N-terminal sequences of *E. coli* TonB and TolA. Residues predicted to lie within the proteins’ transmembrane helices are colored blue; the conserved SHLS motif is indicated by asterisks. The position of the D-box is indicated by a red box. b) Alignment of the TonB D-box with the D-box-like sequence in TolA. Residues predicted by PSIPRED to have high β-strand propensities are italicized. TonB’s pattern of hydrophobic and hydrophilic residues is shown, as in Figure 4; one residue in TolA, Val-53, deviates from this pattern (indicated by arrow). However, in the complex of ExbD with the D-box, the residue in this position faces upward, away from the protein. Hence, there is no reason to think that a hydrophobic residue cannot be accommodated in a complex similar to that observed with ExbD and the TonB D-box.

**References**

1. Weeks, S. D., Drinker, M., and Loll, P. J. (2007) Ligation independent cloning vectors for expression of SUMO fusions. *Protein Expr Purif* **53**, 40-50.

2. Chayen, N. (1997) The role of oil in macromolecular crystallization. *Structure* **5**, 1269-1274.

3. D'Arcy, A., Elmore, C., Stihle, M., and Johnston, J. E. (1996) A novel approach to crystallizing proteins under oil. *Journal of Crystal Growth* **168**, 175-180.

4. Kabsch, W. (2010) XDS. *Acta Crystallogr D Biol Crystallogr* **66**, 125-132.

5. Terwilliger, T. C., Adams, P. D., Read, R. J., McCoy, A. J., Moriarty, N. W., Grosse-Kunstleve, R. W., Afonine, P. V., Zwart, P. H., and Hung, L. W. (2009) Decision-making in structure solution using Bayesian estimates of map quality: the PHENIX AutoSol wizard. *Acta Crystallogr D Biol Crystallogr* **65**, 582-601.

6. Emsley, P., Lohkamp, B., Scott, W. G., and Cowtan, K. (2010) Features and development of Coot. *Acta Crystallogr D Biol Crystallogr* **66**, 486-501.

7. McCoy, A. J., Grosse-Kunstleve, R. W., Adams, P. D., Winn, M. D., Storoni, L. C., and Read, R. J. (2007) Phaser crystallographic software. *J Appl Crystallogr* **40**, 658-674.

8. Sievers, F., Wilm, A., Dineen, D., Gibson, T. J., Karplus, K., Li, W., Lopez, R., McWilliam, H., Remmert, M., Soding, J., Thompson, J. D., and Higgins, D. G. (2011) Fast, scalable generation of high-quality protein multiple sequence alignments using Clustal Omega. *Mol Syst Biol* **7**, 539.

9. McGuffin, L. J., Bryson, K., and Jones, D. T. (2000) The PSIPRED protein structure prediction server. *Bioinformatics* **16**, 404-405.

10. Needleman, S. B., and Wunsch, C. D. (1970) A general method applicable to the search for similarities in the amino acid sequence of two proteins. *J Mol Biol* **48**, 443-453.
